# An Overview of the Putative Structural and Functional Properties of the GHBh1 Receptor through a Bioinformatics Approach

**DOI:** 10.3390/life13040926

**Published:** 2023-03-31

**Authors:** Casper J. H. Wolf, Hanka Venselaar, Marcia Spoelder, Harmen Beurmanjer, Arnt F. A. Schellekens, Judith R. Homberg

**Affiliations:** 1 Department of Psychiatry, Radboudumc, 6525 GC Nijmegen, The Netherlands; 2 Department of Cognitive Neuroscience, Donders Institute for Brain Cognition and Behaviour, Radboudumc, 6525 EN Nijmegen, The Netherlands; 3 Nijmegen Institute for Scientist-Practitioners in Addiction (NISPA), 6525 HR Nijmegen, The Netherlands; 4 Center for Molecular and Biomolecular Informatics, Radboudumc, 6525 GA Nijmegen, The Netherlands; 5 Department of Primary and Community Care, Radboudumc, 6525 GC Nijmegen, The Netherlands; marcia.spoelder-merkens@radboudumc.nl; 6 Behavioural Science Institute, Radboud University, 6525 GD Nijmegen, The Netherlands; 7 Novadic-Kentron Addiction Care, 5261 LX Vught, The Netherlands

**Keywords:** gamma-hydroxybutyric acid, GHB, drug addiction, riboflavin, receptor structure, receptor model

## Abstract

The neurotransmitter γ-hydroxybutyric acid (GHB) is suggested to be involved in neuronal energy homeostasis processes, but the substance is also used as a recreational drug and as a prescription medication for narcolepsy. GHB has several high-affinity targets in the brain, commonly generalized as the GHB receptor. However, little is known about the structural and functional properties of GHB receptor subtypes. This opinion article discusses the literature on the putative structural and functional properties of the GHBh1 receptor subtype. GHBh1 contains 11 transmembrane helices and at least one intracellular intrinsically disordered region (IDR). Additionally, GHBh1 shows a 100% overlap in amino acid sequence with the Riboflavin (vitamin B2) transporter, which opens the possibility of a possible dual-function (transceptor) structure. Riboflavin and GHB also share specific neuroprotective properties. Further research into the GHBh1 receptor subtype may pave the way for future therapeutic possibilities for GHB.

## 1. Introduction

Gamma-hydroxybutyric acid (GHB) is a neurotransmitter that is both a precursor and metabolite for the inhibitory neurotransmitter gamma-aminobutyric acid (GABA). GHB has a high direct affinity for various binding sites in the brain, commonly referred to as the GHB receptor, and a low direct affinity for the inhibitory GABA_B_ receptor [1]. Endogenous GHB occurs in the mammalian brain in concentrations that exclusively activate the GHB receptor and is implicated in energy homeostasis processes on both a micro- (neuronal energy homeostasis) and macro level (sleep–wake homeostasis) [2]. A specific subtype of the rat GHB receptor is primarily located in the hippocampus, frontal cortex, piriform cortex and cerebellum, and to a lesser extent in the striatum, olfactory bulb and thalamus [3]. Low (endogenous) doses of GHB have been shown to increase glutamatergic signaling through GHB receptor activation [4,5,6]. In contrast, exogenous GHB dose-dependently activates the GABA_B_ receptor and is used medically for the treatment of narcolepsy [7]. GHB is also used for recreational purposes due to its dose-dependent euphoric, disinhibiting and sedative properties and may lead to a severe substance use disorder [8,9,10]. Although the structural and functional properties of the GABA_B_ receptor have been extensively studied, there is no clear overview of the structural and functional properties of the GHB receptor.

There are several structures that have a high affinity for GHB (or its analogs NCS-382/Ph-HBTA) [11,12], indicating the existence of several GHB receptor subtypes [13,14]. Recently, a paper was published on a high-affinity target of GHB, identified as the CamKIIα hub domain [15]. However, already in 2002, an incorrectly spliced form of a GHB-binding structure containing eight transmembrane domains was identified through a human genome database search [16]. In 2003, the full-length variant of this GHB-binding structure was first identified as a porcine endogenous retrovirus receptor [17], which was later classified as a (closely related) subtype of the human GHB (GHBh1) receptor. The authors claimed that this full-length protein contained 10 or 11 transmembrane helices [17]. In the same year, Andriamampandry and colleagues cloned a putative rat GHB receptor that had high affinity for GHB but did not correspond with the pharmacological properties or localization of native GHB sites [13,18,19]. Finally, Andriamampandry et al. cloned a GHB receptor (GHBh1) in 2007 using polymerase chain reaction (PCR), based on human frontal cortex cDNA and primers from the putative rat GHB receptor [14]. Through subsequent DNA sequencing and database searches (basic local alignment search tool (BLAST) at NCBI), the GHBh1 receptor was identified as a G-protein coupled receptor 172A, although several studies could not find an activation of G protein by GHB [6,20]. Despite the identification of the GHBh1 receptor, the identity of high-affinity GHB binding sites is still under debate [1,15,19]; it is possible that high-affinity GHB binding sites in the brain consist of multiple subpopulations of various protein structures.

In vitro, it has been established that the GHBh1 receptor is activated by low doses of GHB and NCS-382, a ligand that specifically binds to GHB binding sites [14,21]. Nonetheless, the functional significance of GHBh1 in terms of the pharmacological effects of GHB in humans is unclear and remains speculative. In 2010, Yao et al. classified this structure as a riboflavin transporter (hRFT3), which was suggested to have 10 TM domains [22,23,24]. However, there remains limited knowledge of GHB high-affinity binding sites and the putative GHBh1 in specific. Both the localization and function of the GHBh1 receptor are unknown, and its structural properties are unclear. The aim of the current work is to provide an overview of the structural and functional properties of the GHBh1 receptor using a bioinformatics approach and employing a literature search. Specifically, we will describe the transmembrane structure and intrinsically disordered regions (IDRs) of the GHBh1 receptor and examine the functional overlap between the GHBh1 receptor and the riboflavin transporter. To further explore the possible functional properties of the GHBh1 receptor, we also provide an overview of proteins that may be able to interact with the GHBh1 receptor. Finally, we speculate on the possible clinical role of the GHBh1 receptor.

## 2. Structural Properties of the GHBh1 Receptor

**Figure 1:**
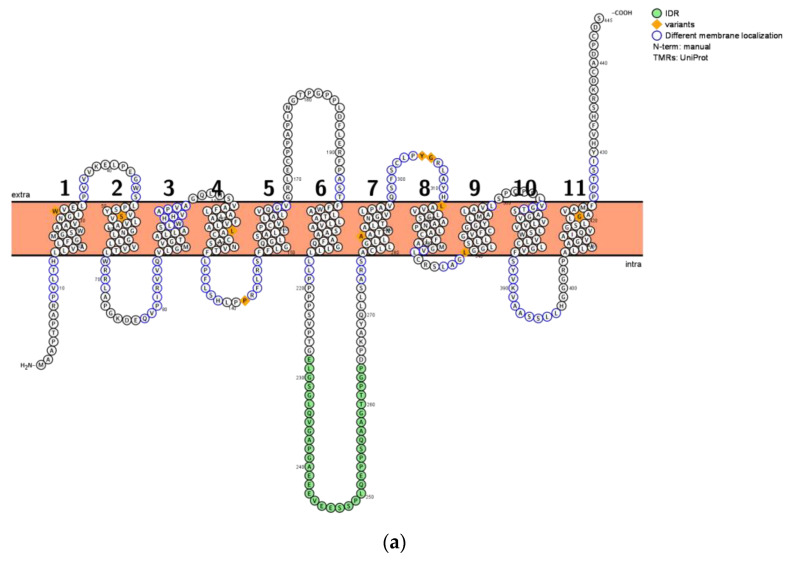

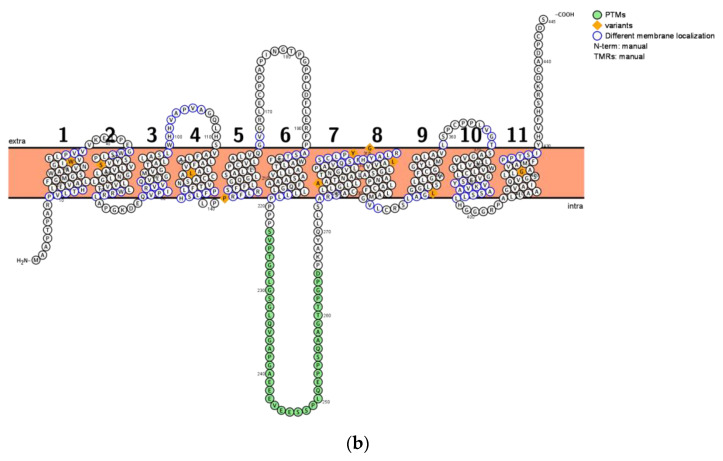
Two-dimensional structure of the GHBh1 receptor based on the (**a**) Uniprot database and (**b**) the Alphafold 2 model, both with an intracellular N-terminus [25]. IDR is highlighted in green. Each letter represents a single amino acid. Differences in TM localization of amino acids are circled in blue. The orange bar represents the cell membrane, and numbers above the cellular membrane represent the number of transmembrane units. Extra = extracellular, intra = intracellular.

We used the amino acid sequence of the GHBh1 receptor, published by Andriamampandry et al. (2007), to assess the putative structure of this receptor (accession code Q9HAB3) [14]. Several predictors, in addition to the Uniprot/Swissprot database, were used to identify various characteristics of the receptor, including the number of transmembrane (TM) helices, terminus orientation and IDRs (see Table 1). Using Protter, an interactive protein visualization tool based on the Uniprot database and Phobius predictor, the two-dimensional receptor structure of the GHB receptor was visualized [25] (Figure 1).

The majority of predictors showed that the GHBh1 receptor is composed of 11 TM helices. Uniprot and two predictors indicated the presence of either one or two IDRs (see Table 1). IDRs are characterized by a large degree of structural adaptability, allowing for the interaction with multiple and structurally diverse ligands. This is in contrast to structured proteins or structured regions that are only able to interact with one specific structure, according to the lock and key principle [26]. All predictors showed an intracellular N-terminus orientation. With this orientation, it is likely that the loop between TM5 and TM6 serves as a ligand binding site, whereas the intracellular loop between TM6 and TM7 might be involved in intracellular signaling cascades. The long extracellular tail containing the C-terminus might also serve as a ligand binding domain. The predictions of the secondary structure of the GHB receptor shown in Table 1 all demonstrate a large overlap with the structure presented in Figure 1.

**Figure 2:**
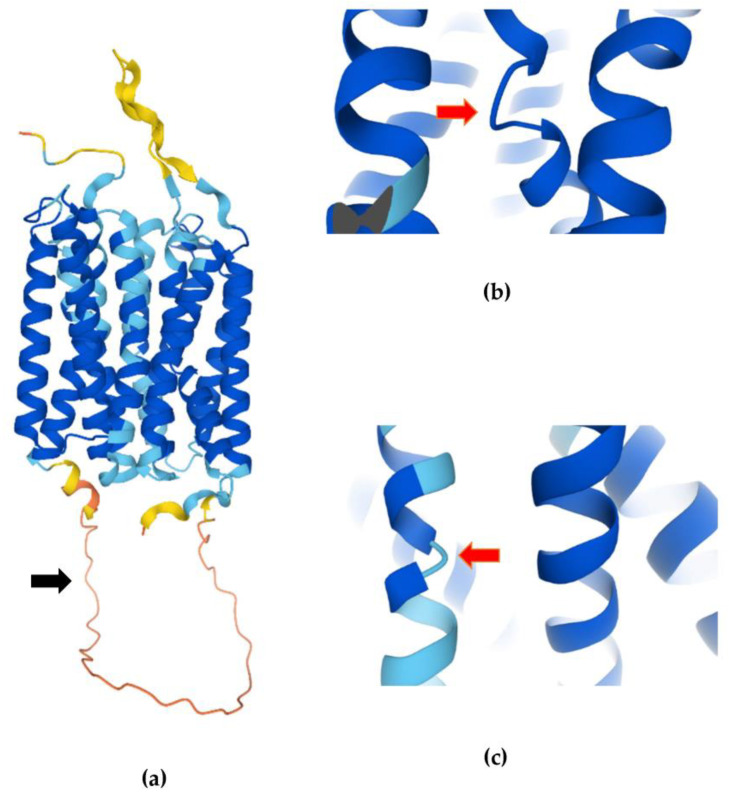
(**a**) Three-dimensional structure of the GHBh1 receptor [27,35]. The loop between TM6 and TM7 is indicated with a black arrow. (**b**) Close-up of the bend present in TM1, indicated with a red arrow. (**c**) Close-up of the bend present in TM2, indicated with a red arrow. Colors indicate per-residue confidence scores ranging from 0–100 (pLDDT). Dark blue: very high (pLDDT > 90), light blue: high (90 > pLDDT > 70), yellow: low (70 > pLDDT > 50), orange: very low (pLDDT < 50). The shown structures were arbitrarily selected from the 3D model to visualize the TM6–TM7 loop, TM1 bend and TM2 bend in the clearest way possible.

In addition to the two-dimensional predictions of the GHBh1, the recently published Alphafold 2 model allows us to also assess the three-dimensional structure of the GHBh1 receptor (Figure 2a) [27]. In accordance with the majority of other predictors, the Alphafold 2 model confirms that the GHB receptor contains 11 transmembrane helices [35]. It appears that the start- and endpoints of the TM helices slightly differ between the Alphafold 2 model and the Uniprot database (Figure 1). This in its turn leads to a difference in the length of some loops between TMs. Alphafold 2 shows very low confidence in the structural prediction of the large loop between TM6–TM7, suggesting that the conformation of these residues will likely not resemble the model and indicating the presence of an IDR (Figure 2a). An IDR between TM6–TM7 is also predicted by Psipred and Predict Protein (Table 1). When we further zoom in on the three-dimensional properties of the receptor, we observe a bend within both TM1 (Figure 2b) and TM2 (Figure 2c). These bends are common in transmembrane helices and are believed to be involved in helix flexibility and conformational changes [36,37]. Overall, the Alphafold 2 model shows primarily overlapping characteristics with other (two-dimensional) predictions of the GHBh1 receptor, and it provides more detailed information on the three-dimensional conformation of the GHBh1 receptor.

## 3. GHB Receptor and Riboflavin Transporter

The GHBh1 receptor amino acid sequence showed a 100% overlap in identity with the Riboflavin transporter sequence, or the *solute carrier family 52, member 2* (*SLC52A2*), also known as human riboflavin transporter 3 (hRFT3) [14,22]. A recent study employed homology modeling with three template structures to construct a three-dimensional model of hRFT3, revealing strong similarities with the Alphafold2 model of the GHBh1 receptor [38].

Riboflavin, also known as vitamin B2, is a water-soluble micronutrient that is not naturally present in the human body but can be obtained through the intestinal absorption of several food products, such as dairy products, meat, fish, fruits and vegetables [39]. Following intestinal absorption, riboflavin is eventually transported from the extracellular environment into the cell through a membrane transport protein (or transporter). Riboflavin is thought to be involved in a variety of cellular energy homeostasis processes in peripheral and brain tissue. More specifically, it plays a vital role in several pathways in mitochondria, such as oxidative phosphorylation of the electron transport chain, the Kreb’s cycle and the tricarboxylic acid (TCA) cycle. These key pathways are involved in the production of ATP, responsible for membrane stability and energy-related cellular functions [39,40].

The hRTF3 structure is primarily present in the brain and salivary glands and has been implicated in neurological disorders such as spinocerebellar ataxia and Brown-Vialleto-Van Laere (BVVL) syndrome [41]. hRFT3 and has been previously characterized as both a 10 and 11 TM structure [42,43]. hRFT3 can also bind, but not transport, the riboflavin co-enzymes Flavin Adenine Dinucleotide (FAD) and Flavin Mononucleotide (FMN) with low affinity [42]. FMN, in addition to lumiflavin and Mg^2+^, are able to inhibit riboflavin binding and transport [44]. Riboflavin transport through hRTF3 is suggested to be Ca^2+^ dependent [44]. hFRT3 has a strong homology with other riboflavin transporters, including hRFT1 and hRFT2, which are primarily present in the placenta/small intestine and testis/small intestine/prostate, respectively [42].

At first sight, it is surprising that a GHB receptor and a riboflavin transporter consist of an identical amino acid sequence, since transporters and receptors strongly differ in functionality. A transporter mediates the uptake of a specific ligand by the cell, after which the ligand itself can act as an intracellular signaling molecule. In contrast, a receptor interacts with an external ligand, leading to a conformational change of the receptor and inducing downstream intracellular processes. However, there is evidence for the existence of a structure functionally linking transporters and receptors, called a transceptor [45].

The first evidence of the concept of transceptors was presented by Johan Thevelein at a conference in Spain in 1999 (and later published by his group in 2003), where he showed evidence of a cellular sensing mechanism (Gap1) that operates through transporters in *Saccharomyces cerevisiae* [46]. Transceptors combine both transporter and receptor functions, while these two functions are generally independent from each other. Known transceptors include structures specific for phosphate (Pho84), ammonium (Mep2), sulfate (Sul1 and Sul2), iron (Ftr1) and zinc (Zrt1). Transceptors are usually highly induced upon micronutrient starvation and are downregulated by substrate-induced endocytosis [45].

Transceptors generally have partially overlapping binding sites in the same substrate-binding pocket. The subsequent effect depends on the specific binding ligand. The IDR between TM6 and TM7 might play a crucial role in the ability of the GHB/riboflavin transceptor to mediate the dual function of the structure. Figure 1 shows that the IDR between TM6 and TM7 is likely situated within the cytoplasm, as are the majority of transmembrane IDRs [47,48]. The intracellular orientation of the IDR makes the role of a ligand-binding site unlikely but indicates a role of the IDR in the flexible initiation of intracellular signaling cascades. Upon interaction of the IDR with an intracellular compound, the IDR might form a stable structure around the compound. When multiple compounds are bound to the IDR, it may even mediate and accelerate the interaction between these ligands [49].

Intracellular IDRs have been implicated in the regulation of transmembrane protein function and activity. These IDRs are likely involved in the recruitment of proteins that are activated by signaling molecules, such as kinases and phosphatases [50]. One hypothesis could be that the extracellular binding ligand determines which intracellular proteins are bound to the IDR and consequently determines which intracellular pathways are triggered upon transceptor activation.

The question remains how both GHB and riboflavin, which are two structurally distinct compounds, can bind to one structure. It is possible that the C-terminus tail may also act as a ligand binding site, since it has been shown that the deletion of the C-terminal region of hRFT3 leads to impairment of transport function [23]. However, in this study, the C-terminal was predicted to be situated in the cytoplasm, hampering direct translation of the results. Future studies should examine the binding characteristics of GHB and riboflavin at the transceptor and experimentally confirm the binding site localization and dual function of this structure.

## 4. Functional Properties of GHBh1 Binding Proteins

Although the existence of a dual-function binding site for GHB and riboflavin has not been experimentally verified, GHB and riboflavin independently show overlapping functional properties. Riboflavin and GHB are both suggested to have neuroprotective properties under energy-deprived circumstances, such as oxygen deprivation (ischemia). It has been shown that riboflavin protects tissue against ischemia-reperfusion injury in the brain [51,52,53]. Riboflavin, in combination with other vitamin supplements, has also been shown to lead to improvement in Erythrocyte Glutathione Reductase (EGR) during alcohol withdrawal, increasing levels of the anti-oxidant glutathione and decreasing oxidative stress [54]. Similar to riboflavin, GHB protects against ischemia-reperfusion damage through improved cerebral blood flow, cerebral vascular density, and improved pCO_2_ and pO_2_ in the ischemic region [55]. It has been shown that GHB can exert its neuroprotective effects following ischemia through CamKIIα activation [15]. Although GHB is used as prescription medication, it is unknown whether GHB is able to (indirectly) influence antioxidant activity. The role of the GHBh1 receptor in the neuroprotective properties of GHB and riboflavin remains elusive.

In order to better understand the functions of the GHBh1 receptor, we can look to other proteins that may bind the GHBh1 receptor. Using the publicly available database Biological General Repository for Interaction Datasets (BioGRID) [56], we summarized a list of proteins with known functions that are suggested to interact with the GHBh1 receptor (Table 2). We did not show interactors that were identified based on genetic interactions, nor structures that interacted with possible downstream proteins of the GHBh1 receptor. Table 2 shows putative interactors identified through a protein-fragment complementation assay (PCA), affinity capturing, or a combination of transcriptional activation domain (TAD) + DNA binding domain (DBD) interaction.

The putative GHBh1 receptor interactors can be roughly divided into three classes: interactors involved in the immune response (SPPL2B, TRIM25), proteins involved in the cell cycle (CDC23, UPK1A) and mitochondrial proteins (ATP13A1 and GHITM), which is in line with the key role of riboflavin in several mitochondrial pathways. The functional implications of other interactors are not intuitive, based on our current knowledge of GHB, riboflavin or the GHBh1 receptor, but may encourage further exploration of these protein interactions.

## 5. Clinical Implications and Limitations

To speculate on the role of GHBh1 in the pharmacological effects of GHB in humans, we can look at the cellular response characteristics of GHBh1. GHB patch clamping experiments have shown that activation of the GHBh1 by a low dose of GHB leads to a clear cellular response, which is rapidly followed by a prolonged period of desensitization [14]. This indicates slow recovery kinetics following desensitization or internalization, as is also often observed with transceptors upon repeated substrate binding [45]. The high affinity of GHB for GHBh1, in combination with the rapid and long desensitization of the receptor, make it improbable that the GHBh1 receptor is involved in the (GABAergic) response following high, exogenous doses of GHB. Considering the use of high doses of GHB in patients with GHB use disorder (GUD), it is unlikely that GHBh1 receptor activation plays a significant role in the characteristics of GUD (such as comas, severe withdrawal symptoms or high relapse rates). However, the GHBh1 receptor may play a role in the cellular effects of exogenous GHB. Opposing results have been reported on the cellular effects of exogenous GHB. On the one hand, there are indications that GHB use leads to neuronal damage and cognitive deterioration [62,63,64]. On the other hand, GHB is safely used as a treatment for the neurological disorder narcolepsy and has been tested for the treatment of alcohol use disorder [65,66]. Future studies should examine the functional significance of GHBh1 in terms of the pharmacological effects of GHB in humans.

The findings and interpretations presented in this opinion article should be considered in the light of some limitations. In this paper, we discussed the GHBh1 amino acid sequence as retrieved by Andriamampandry et al. (2007) as a high-affinity GHB receptor [14]. It should be noted that other subtypes of the GHB receptor exist, such as C12K32 and CamKIIα, which will exhibit distinct structural and functional characteristics [14]. This opinion article solely addresses the GHBh1 subtype, and our findings are likely not to be translatable to other GHB receptor subtypes. Additionally, the GHBh1 subtype has not been verified as a native GHB receptor. Although the GHBh1 receptor has been shown to bind low doses of GHB and the GHB receptor-specific antagonist NCS-382, it should be further examined whether the GHBh1 receptor shows overlap with native GHB sites.

Despite the large overlap in results between predictors, some discrepancy can be found regarding the number of predicted TM helices. Phobius and TM-pred predicted 10 transmembrane helices with high certainty. It appears that TM-pred does not predict a TM helix within the region of 81–103, while Phobius does not predict a TM helix within the region of 14–34. Instead, Phobius predicts a long extracellular amino acid chain that includes a signaling peptide chain at the N-terminus. Nevertheless, the majority of predictors, including the Alphafold 2 model, show a presence of 11 TM domains and a large overlap in their outcomes, providing a solid basis for the findings that are presented in this opinion article. Future studies should experimentally verify the exact structure of the GHBh1 receptor in order to further understand the functional mechanism of this structure.

In conclusion, the GHBh1 receptor contains 11 transmembrane units and at least one IDR. The amino acid sequence of the GHBh1 receptor is identical to the sequence of the riboflavin transporter, indicating the existence of a dual-function GHB/riboflavin transceptor. Proteins involved in the cell cycle, immune response, and mitochondrial processes may also interact with the GHBh1 receptor. This research provides a theoretical framework regarding the functional properties of the GHBh1 receptor, allowing experimental verification of our findings, which may lead to a further understanding of the possible (neuro)biological role of the GHBh1 receptor.

## Figures and Tables

**Table 1:** Overview of the number of transmembrane helices, terminus orientation and IDRs assessed by various predictors and the Uniprot/Swissprot database.

Predictor/Database	Transmembrane Helices	N-Terminus Intra- Cellular/Extracellular	Intrinsically Disordered Regions
Alphafold 2 [27]	11	NA	NA
Phobius [28]	10	Extracellular	NA
Predict Protein [29]	11	NA	1 (intracellular, 237–261, between TM6 and TM7)
Psipred [30,31]	11	Intracellular	2 (intracellular, 1–11, before TM 1, and 223–270, between TM6 and TM7)
MEMSAT 3 [32]	11	Intracellular	NA
TMHMM [33]	11	Intracellular	NA
TM-pred [34]	10	Intracellular	NA
Uniprot [24]	11	NA	1 (intracellular, 228–264, between TM6 and TM7)

**Table 2:** Overview of possible interactors of the GHBh1 receptor structure [56].

Interactor	Description	Function
SPPL2B [57,58]	Signal peptide peptidase like 2B	Involved in immune response by cleaving TNFα in dendritic cells
ATP13A1 [58]	ATPase type 13A1	Mediates removal/extraction of mislocalized mitochondrial transmembrane proteins from the endoplasmic reticulum membrane
CDC23 [59]	Cell division cycle 23	Part of a ubiquitin ligase that controls progression through mitosis
FAM209A [60]	Family with sequence similarity 209, member A	May play a role in sperm acrosome biogenesis
GHITM [58]	Growth hormone inducible transmembrane protein	Plays a role in apoptosis through mediating mitochondrial morphology and cytochrome c release
TRIM25 [61]	Tripartite motif containing 25	Ubiquitin ligase regulating the innate immune response
UPK1A [57]	Uroplakin 1A	Mediating signal transduction events involved in regulating cell development, activation, growth and motility

## Data Availability

No new data were created in this study.
